# Natural Products in the Metabolic and Endocrine Modulation of Polycystic Ovary Syndrome: Current Perspectives

**DOI:** 10.3390/nu18060964

**Published:** 2026-03-18

**Authors:** Siqi Liu, Rui Wang, Weili Yu, Chuanjing Shi, Xi Wang, Aifen Liu, Lei Zhang

**Affiliations:** 1 Institute of Interdisciplinary Integrative Medicine Research, School of Medicine, Nantong University, Nantong 226001, China; 2 Department of Pharmaceutical Botany, School of Pharmacy, Naval Medical University, Shanghai 200433, China

**Keywords:** PCOS, natural products, endocrine disorders, insulin resistance, epigenetic regulation, gut microbiota

## Abstract

Polycystic ovary syndrome (PCOS) is a prevalent endocrine and metabolic disorder, primarily characterized by reproductive dysfunction, insulin resistance (IR), and long-term metabolic complications. Current first-line pharmacological treatments, including oral contraceptives, anti-androgens, and insulin sensitizers, can alleviate clinical symptoms but often fail to fully address the underlying pathophysiology, and their long-term use is frequently limited by adverse effects. Natural products, owing to their multi-target regulatory properties and favorable safety profiles, have emerged a promising adjuvant therapeutic strategy. This review systematically summarizes how natural products exert beneficial effects through mechanisms such as improving metabolic homeostasis by enhancing insulin sensitivity and mitigating oxidative stress and chronic inflammation; restoring endocrine balance by modulating the hypothalamic–pituitary–gonadal axis to reduce hyperandrogenemia and promote ovulation; and utilizing emerging pathways including regulating gut microbiota homeostasis and epigenetic modifications as a novel avenue for PCOS drug development. Preclinical and clinical evidence collectively indicates that these agents hold significant translational potential to ameliorate metabolic disturbances and improve reproductive outcomes, providing a scientific foundation for future integrated intervention strategies in PCOS.

## 1. Introduction

Polycystic ovary syndrome (PCOS) is one of the most prevalent endocrine–metabolic disorders, affecting approximately 5–18% of reproductive-aged women worldwide [1]. The condition manifests through a heterogeneous constellation of symptoms, including oligo-anovulation, menstrual irregularities, clinical or biochemical hyperandrogenism, and infertility, collectively imposing significant physical and psychological burdens on affected individuals [2]. Importantly, PCOS is strongly associated with significant metabolic disturbances, such as insulin resistance (IR) and dyslipidemia, which elevate the long-term risk for type 2 diabetes and cardiovascular morbidity [3,4]. Diagnosis, according to the 2023 International Guidelines, is based on meeting at least two of the following three criteria, after exclusion of other etiologies: clinical or biochemical hyperandrogenism, ovulatory dysfunction, or polycystic ovarian morphology on ultrasound [5,6]. While diagnostic methods are well established, effective long-term treatment of PCOS remains a major clinical challenge, underscoring an urgent need for more effective and mechanism-based therapeutic strategies [7].

Contemporary management of PCOS primarily focuses on alleviating specific symptoms, often with limitations. Combined oral contraceptives (COCs), as first-line therapy, regulate menstrual cycles and mitigate hyperandrogenism but carry risks of thromboembolism, hypertension, and cardiovascular events [8,9]. Metformin improves metabolic parameters as an insulin sensitizer [10], but its use is limited by gastrointestinal side effects, variable efficacy, and insufficient evidence as a monotherapy on the core reproductive features like ovulation and hirsutism [11]. Therefore, safer, multi-targeted, and more personalized therapeutic options are urgently needed. Lifestyle and dietary interventions should serve as the cornerstone of a personalized, multi-step approach for PCOS. A meta-analysis reported that a diet rich in fiber and with a low glycemic load improves health outcomes in PCOS patients [12]. Current recommendations emphasize that regular physical activity optimizes hormonal and metabolic profiles, in addition to supporting weight management [13]. Although no single dietary approach is universally endorsed, low-glycemic-index foods combined with a high fiber intake are generally recommended for hyperinsulinemia [13]. Thus, an effective nutritional strategy for PCOS should be multifaceted and goal-oriented, as targeted dietary modifications lead to better biochemical and metabolic outcomes than general advice alone [14].

Natural products, including bioactive compounds derived from plants, herbs, and whole foods, represent a promising category of nutrients for PCOS management due to their multi-targeted therapeutic potential and favorable safety profile. Emerging evidence demonstrates this due to their pleiotropic effects. Preclinical and clinical studies indicate that natural products such as quercetin, resveratrol, berberine, curcumin, and rutin can exert pleiotropic effects, including enhanced insulin sensitivity, reduced oxidative stress, mitigated chronic inflammation, and modulated hormonal activity. Mechanistically, they target the key signaling pathways involved in PCOS, such as the activation of AMP-activated protein kinase (AMPK), modulation of CaMKIIβ and peroxisome proliferator-activated receptor gamma (PPARγ), suppression of NF-κB, and activation of Sirtuin 1 (SIRT1) [15,16,17,18]. This multifaceted regulation renders natural products promising agents for a more integrated PCOS treatment paradigm.

Therefore, this review aims to provide a comprehensive synthesis of the current knowledge on natural products in PCOS, with emphasis on their regulatory effects on metabolic-endocrine profiles as reflected by IR, oxidative stress, apoptosis, epigenetic modifications, and gut microbiota dysbiosis. By integrating mechanistic insights with experimental and clinical evidence, we highlight their potential role as complementary or alternative therapeutic strategies, paving the way toward safer, personalized, and mechanism-based management of PCOS.

## 2. Pathophysiology of PCOS and Therapeutic Potential of Natural Compounds

PCOS is not a single-pathway disorder but rather a multifaceted condition in which reproductive and metabolic abnormalities are tightly interwoven. At the core of its pathophysiology lies a complex interplay between endocrine dysregulation, metabolic disturbances, and cellular stress responses. These mechanisms converge to create a hyperandrogenic and insulin-resistant milieu that disrupts ovarian folliculogenesis, impairs ovulation, and drives systemic metabolic dysfunction. Recent advances further highlight the contribution of oxidative stress, epigenetic alterations, and gut microbiota dysbiosis, underscoring PCOS as a syndrome shaped by both intrinsic genetic susceptibility and extrinsic environmental influences. Understanding these interconnected processes is essential for elucidating the disease mechanism and identifying novel therapeutic targets, including the use of natural products with multi-level regulatory potential.

### 2.1. Targeting Hyperandrogenism and HPO Axis Dysregulation in PCOS

**Figure 1:**
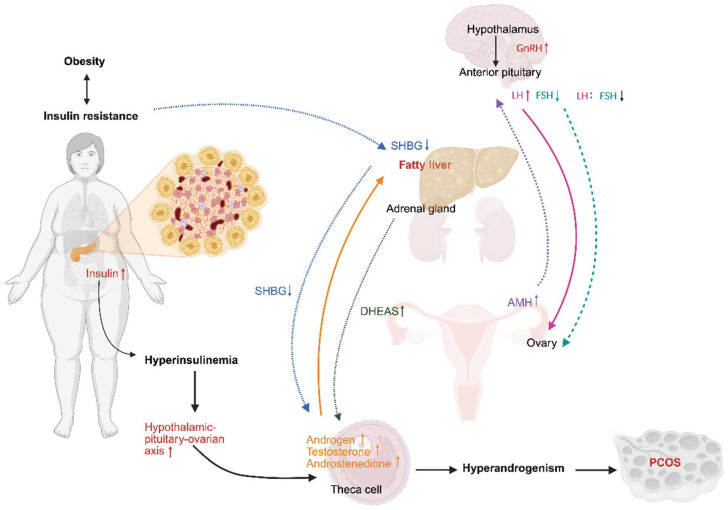
Mechanisms linking obesity to female reproductive dysfunction. Obesity-driven IR and hyperinsulinemia disrupt the HPO axis, leading to reduced hepatic SHBG, increased ovarian/adrenal androgen production, and altered GnRH pulsatility with an elevated luteinizing hormone LH/FSH ratio. These changes reinforce ovarian androgen excess, impair folliculogenesis, and are reflected by elevated AMH. Abbreviations: HPO, hypothalamic–pituitary–ovarian; SHBG, sex hormone-binding globulin; DHEAS, dehydroepiandrosterone sulfate; GnRH, gonadotropin-releasing hormone; LH, luteinizing hormone; FSH, follicle-stimulating hormone; AMH, anti-Müllerian hormone. Symbols: ↑ indicates an increase; ↓ indicates a decrease. Arrows: Blue dashed (↓ SHBG → ↑ free androgens); Orange solid (↑ ovarian androgens → ↑ fatty liver); Green dashed (adrenal gland → ↑ DHEAS); Purple dashed (↑ AMH → ↑ LH); Magenta solid (↑ LH); Teal dashed (↓ LH:FSH ratio); Black solid (obesity → insulin resistance → hyperandrogenism → PCOS). This figure was created in BioRender. Eva, L. (2026) https://BioRender.com/wj0ioqr (accessed on 23 February 2026).

The pathogenesis of PCOS is fundamentally rooted in a self-amplifying cycle of hypothalamic–pituitary-ovarian (HPO) axis dysregulation and hyperandrogenism [19,20]. At the central level, altered gonadotropin-releasing hormone (GnRH) pulsatility elevates the luteinizing hormone (LH)-to-follicle-stimulating hormone (FSH) ratio, a clinical hallmark of ovulatory dysfunction [21]. Elevated LH levels overstimulate theca cells, boosting enzymes like CYP17A1 and CHP11A1, which increases circulating androgens including 17-hydroxyprogesterone, testosterone, and androstenedione [22,23], clinically manifesting as hirsutism, acne, or alopecia. Concurrently, insufficient FSH weakens granulosa cell function and reduces aromatase CYP19A1 activity, thereby limiting the conversion of androgens into estrogens [20,24]. These disruptions result in follicular maturation arrest, increased atresia, and anovulation, confirmed by polycystic ovarian morphology on ultrasound. Peripheral IR further exacerbates this state as hyperinsulinemia synergizes with LH to further potentiate ovarian androgen biosynthesis and suppresses hepatic production of sex hormone-binding globulin (SHBG), thereby increasing the bioavailability of free testosterone [25], as reflected by clinical assessments of fasting insulin, HOMA-IR, and the free androgen index. This creates a vicious cycle where hyperandrogenism itself can give feedback to enhance GnRH pulsatility. Therefore, GnRH antagonists potentially offer a therapeutic avenue [26] (Figure 1).

Natural products intervene in this cycle through multi-targeted mechanisms, addressing both central regulatory defects and peripheral steroidogenic excess [27]. To modulate the HPO axis, compounds like resveratrol can attenuate aberrant GnRH neuronal activity, helping to normalize gonadotropin secretion [28,29]. At the ovarian level, quercetin suppresses androgen synthesis by inhibiting enzymes like CYP17A1, while berberine enhances the conversion of androgens to estrogens via CYP19A1 upregulation [30,31,32]. Others include ginsenosides, which support granulosa cell health and enhance aromatase activity, promoting follicular development [33]. Furthermore, several natural compounds can upregulate SHBG production, thereby reducing circulating free androgen levels, and some exhibit potential for AR antagonism, blocking the final pathway of androgen action. This coordinated approach simultaneously targets the upstream drivers and downstream effects of hyperandrogenism, aligning with the complex endocrine pathophysiology of PCOS. Table 1 lists the distinct natural products targeting hormonal dysregulation in PCOS.

### 2.2. Targeting IR in PCOS

**Figure 2:**
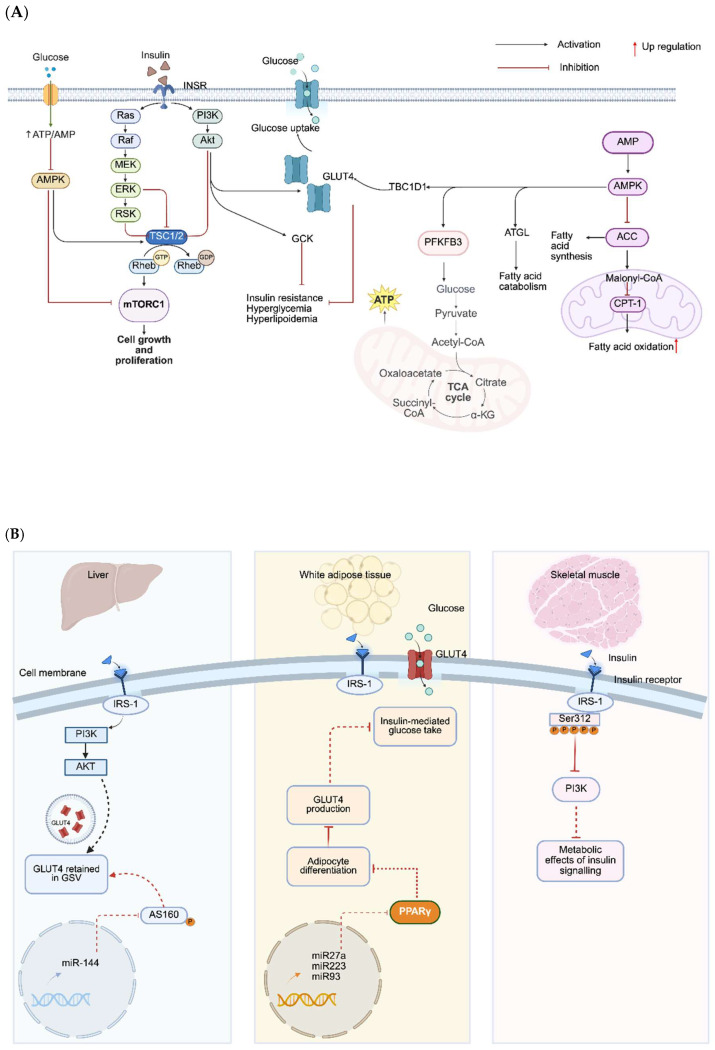
Tissue-Specific dysregulation of insulin signaling in PCOS. (**A**) Canonical insulin signaling pathway and its metabolic consequences. The core insulin signaling cascade and its downstream metabolic effects with mechanisms of regulation, canonical insulin signaling through PI3K–Akt that promotes GLUT4 translocation, glycogen synthesis via GSK3 inhibition, and mTORC1-driven growth, as well as AMPK-mediated restraint of mTORC1; dysfunction of this network produces hyperglycemia through impaired uptake and increased gluconeogenesis, hyperlipidemia through increased fatty acid synthesis and reduced oxidation via ACC/CPT-1, and diminished mitochondrial substrate flux through the TCA cycle. Key: → activation; ⊣ inhibition. (**B**) Tissue-specific molecular defects in PCOS, including liver miR-144-driven downregulation of IRS-1 and AS160 that impairs Akt signaling and GLUT4 vesicle trafficking; white adipose tissue defects in insulin-mediated glucose uptake due to impaired GLUT4 translocation/production and altered adipocyte differentiation influenced by miR-27a, miR-223, and miR-93; and skeletal muscle hyperinsulinemia and inflammation-induced inhibitory serine phosphorylation of IRS-1 (e.g., Ser312) that blunts PI3K–Akt signaling and the metabolic actions of insulin. Abbreviations: ACC, acetyl-CoA carboxylase; AMPK, AMP-activated protein kinase; AS160, AKT substrate of 160 kDa; CPT-1, carnitine palmitoyltransferase I; GLUT4, glucose transporter type 4; GS, glycogen synthase; GSV, GLUT4 storage vesicles; GSK3, glycogen synthase kinase 3; IRS-1, insulin receptor substrate 1; miR, microRNA; mTORC1, mechanistic target of rapamycin complex 1; PI3K, phosphoinositide 3-kinase; TCA, tricarboxylic acid; TSC1/2, tuberous sclerosis complex 1/2. Arrows: → indicates activation; ⊣ indicates inhibition; ↑ indicates up-regulation. Arrow colors are solely for matching corresponding content to improve clarity and visual consistency. (**A**) was created in BioRender. Eva, L. (2026) https://BioRender.com/mp6ntpf (accessed on 23 February 2026). (**B**) was created in BioRender. Eva, L. (2026) https://BioRender.com/4ynplb3 (accessed on 23 February 2026).

IR, affecting nearly two-thirds of women with PCOS, is a central pathophysiological mechanism often accompanied by compensatory hyperinsulinemia [54,55]. In the liver, aberrant serine phosphorylation of IRS-1/IRS-2 and impaired PI3K/Akt signaling disrupt insulin-mediated suppression of gluconeogenesis, leading to excessive hepatic glucose output and dyslipidemia [56,57,58]. In adipose tissue, reduced GLUT4 expression, unrestrained lipolysis, and abnormal adipokine secretion elevate circulating free fatty acids (FFAs) and promote chronic inflammation, worsening systemic IR [59,60]. In skeletal muscle, constitutive activation of MAPK pathways promotes inhibitory IRS-1 serine phosphorylation, selectively impairing PI3K/Akt-mediated GLUT4 translocation and glucose uptake [57,61,62]. These tissue-specific defects are reinforced by elevated FFAs, pro-inflammatory cytokines, and oxidative stress, which establish the hyperinsulinemia environment that drives hyperandrogenism, disrupts the HPO axis, and perpetuates the PCOS phenotype (Figure 2).

Multiple natural products have demonstrated therapeutic potential for PCOS by targeting these IR mechanisms through diverse molecular pathways (Table 2). For instance, berberine enhances insulin sensitivity via AMPK activation and IRS-1 modulation [63]. Resveratrol exerts beneficial metabolic effects through SIRT1/AMPK-mediated anti-inflammatory and antioxidative actions [64]. Quercetin improves IR by inhibiting the NF-κB pathway and reducing inflammatory cytokines [30,65]. Comparable with metformin, inositol (myo- and D-chiro-inositol) demonstrates insulin-sensitizing and favorable safety profiles in PCOS [39,66]. Emerging evidence also supports that ginsenosides improve fat metabolism and reduce IR [67]. Collectively, natural products can target the tissue-specific defects underlying PCOS-associated IR, supporting their potential as adjunctive or alternative therapeutic strategies.

### 2.3. Protective Effects of Natural Products Against Multi-Cellular Damage in PCOS

In PCOS, oxidative stress serves as a central pathological driver, linking hyperandrogenism, IR, and chronic inflammation to downstream cellular damage. Excessive reactive oxygen species (ROS) compromise mitochondrial and endoplasmic reticulum (ER) homeostasis, leading to ER stress within the follicular microenvironment [88,89]. Consequently, ER stress acts as a pivotal mediator by activating apoptotic cascades and facilitating ferroptosis, which has been confirmed in PCOS cell models [90,91]. Furthermore, studies on granulosa cells emphasize that oxidative stress activates multiple signaling pathways, including PI3K/Akt, MAPK, and NF-κB, which converge upon ER stress and subsequent programmed cell death [92].

Apoptosis, a regulated form of programmed cell death, is upregulated in ovarian granulosa cells, driving follicular atresia and impairing ovarian function in PCOS [93,94,95]. Specifically, the p53 signaling pathway enhances the expression of pro-apoptotic genes such as BAX, FAS, and CASP8 in human granulosa cells from PCOS patients [96]. Additionally, elevated levels of LNK in PCOS inhibit the AKT/FOXO3 survival pathway, promoting granulosa cell apoptosis in an IR-dependent manner [97].

Ferroptosis, an iron-dependent form of programmed cell death characterized by glutathione peroxidase 4 (GPX4) suppression and acyl-CoA synthetase long-chain family member 4 (ACSL4) activation, has emerged as a critical contributor to ovarian dysfunction. This process is notably prominent in ovarian granulosa cells, where ER stress and oxidative injury synergistically trigger ferroptosis pathways [90]. Elevated serum ferritin levels and ROS further corroborate the role of iron-mediated oxidative damage in PCOS-associated ferroptosis [98]. Inhibition of ACSL4 has been proposed as a therapeutic avenue to counteract ferroptosis [99], with altered expression of GPX4, transferrin, and ferritin serving as potential biomarkers in PCOS-associated infertility [100]. Additionally, ferroptosis-related EGLN1 levels were elevated in the granulosa cells and plasma of PCOS patients, as well as in the plasma and ovaries of a PCOS mouse model. The EGLN1-HIF1α-ferroptosis axis subsequently validated the therapeutic effect in PCOS mice [101]. Furthermore, dipeptidyl Peptidase 4 (DPP4)-driven ferroptosis exacerbates PCOS-associated endometrial dysfunction, while its suppression enhances stromal cell decidualization capacity and implantation potential, supporting sitagliptin intervention in PCOS patients to enhance reproductive outcomes including higher clinical pregnancy and live birth rates [102].

**Figure 3:**
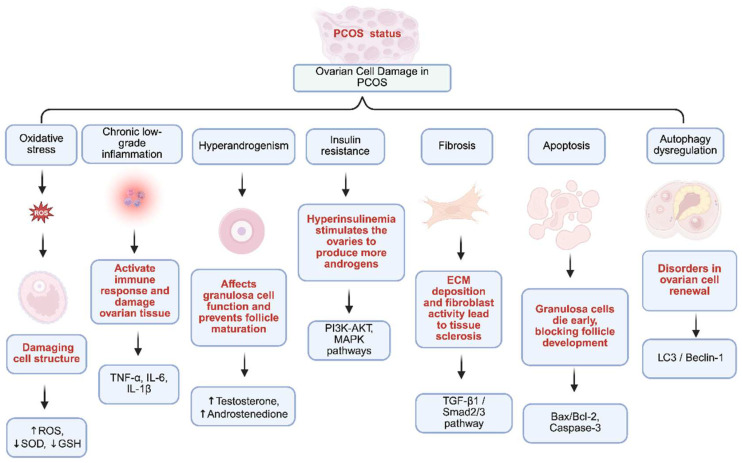
Proposed mechanisms of ovarian cell damage in PCOS. The key pathophysiological drivers and their interconnected consequences leading to impaired ovarian function in PCOS, integrating oxidative stress and chronic low-grade inflammation (↑ TNF-α, ↑ IL-6, ↑ IL-1β) with hyperandrogenism (↑ testosterone, ↑ androstenedione) and IR-driven dysregulation of PI3–Akt and MAPK signaling to produce multifaceted ovarian injury; downstream consequences include fibrosis via TGF-β1/Smad2/3-mediated extracellular matrix deposition, apoptosis via Bax/Bcl-2 imbalance and Caspase-3 activation, and autophagy dysregulation marked by altered LC3 and Beclin-1, collectively damaging granulosa cell function, impairing follicular maturation, and promoting follicular arrest. Abbreviations: ECM, extracellular matrix; IL, interleukin; LC3, microtubule-associated proteins 1A/1B light chain 3B; TGF-β1, transforming growth factor beta 1; TNF-α, tumor necrosis factor-alpha. Arrows: **↑**, increase; **↓**, decrease. This figure was created in BioRender. Eva, L. (2026) https://BioRender.com/x3wipm4 (accessed on 23 February 2026).

Apoptosis and ferroptosis in PCOS ovarian granulosa cells not only lead to cell loss but also amplify inflammation. Both forms of programmed cell death facilitate the release of damage-associated molecular patterns (DAMPs), which subsequently activate NF-κB signaling to drive a self-perpetuating cycle of oxidative damage, IR, and ovarian dysfunction. This mechanism is further supported by evidence that miR-93-5p promotes both apoptosis and ferroptosis in granulosa cells via NF-κB activation, and that DAMPs can initiate NF-κB-mediated sterile inflammation in ovarian and reproductive tissues [100,103] (Figure 3).

Therapeutic strategies targeting the cellular stress–cell death axis in PCOS are gaining traction. *N*-acetylcysteine (NAC) enhances glutathione, reduces oxidative stress, improves insulin sensitivity, and boosts pregnancy rates and ovulation efficacy in PCOS models [104,105]. ER stress inhibition via compounds such as TUDCA suppresses apoptosis and normalizes related gene expression [106]. Moreover, natural bioactive compounds, including resveratrol, curcumin, quercetin, EGCG, and formononetin, modulate key inflammatory and metabolic signaling pathways such as NF-κB, Nrf2, SIRT, and AMPK, resulting in antioxidant, anti-inflammatory, and metabolic benefits [107,108,109] (Table 3). In summary, oxidative stress initiates a pathological cascade amplified by ER stress and executed via apoptosis and ferroptosis. Together, this interconnected “stress–death axis” highlights the potential of antioxidant and epigenetic interventions for both reproductive and metabolic dysfunctions in PCOS.

### 2.4. Protective Effects of Natural Products Against Epigenetic Regulation in PCOS

Epigenetic regulation serves as a mechanistic bridge between genetic predisposition and environmental factors, such as hyperandrogenism and IR, in PCOS [132,133]. The three principal epigenetic mechanisms include DNA methylation, histone post-translational modifications, and non-coding RNAs, and together, they modulate gene expression without altering the DNA sequence. These mechanisms are dynamic, reversible, and highly responsive to environmental stimuli, thereby contributing to the sustained reproductive and metabolic dysfunction characteristic of the syndrome [134].

DNA methylation is a key epigenetic mechanism in PCOS, with patients exhibiting altered methylation patterns in their blood compared to healthy controls. These changes contribute to the transgenerational transmission of PCOS traits and offer potential diagnostic markers and therapeutic targets [135]. Aberrant methylation disrupts genes critical for ovarian function and metabolism. In granulosa cells, hypomethylation of the LHCGR promoter enhances LH sensitivity and promotes hyperandrogenism [136]. Meanwhile, hypermethylation of the insulin receptor (INSR) reduces insulin receptor expression and exacerbates IR [137]. Additionally, hypomethylation of *PPARG* leads to its overexpression in ovarian cells, implicating lipid dysfunction [138], and moreover, hypermethylation of PPARG1 genes, linked to mitochondrial dysregulation, is strongly associated with combined IR and hyperandrogenism in PCOS [139]. Notably, caloric restriction prevents PCOS inheritance through oocyte-mediated DNA methylation reprogramming, underscoring the potential for nutritional interventions to modulate epigenetic risk [140]

Histone post-translational modifications (HPTMs) are key epigenetic regulators that influence gene expression, chromatin structure, and metabolism. In PCOS, HPTMs contribute to disease pathogenesis [141]. In granulosa cells, elevated H3K27me3 at the CYP19A1 promoter is associated with reduced aromatase expression and impaired androgen–estrogen conversion [142]. In PCOS follicles, enhanced fatty acid utilization and altered amino acid metabolism induce oxidative stress and H3K27me3 enrichment in oocytes, compromising maturation [134,143].

In addition, non-coding RNAs play a crucial role. Multiple miRNAs, including miR-93, miR-21, and miR-223, suppress IRS1 and impair insulin signaling. lncRNA-H19 upregulates CYP17A1 to promote excessive androgen synthesis, while reduced expression of circRNA_0043533 induces granulosa cell cycle arrest [142,144]. Simultaneously, oxidative stress-induced miR-181a activation decreases acetylation of FOXO1, promoting granulosa cell apoptosis and accelerating follicular atresia [145]. Together, these epigenetic mechanisms converge to disrupt ovarian function, insulin sensitivity, and metabolic homeostasis, thereby reinforcing the complex endocrine and reproductive abnormalities characteristic of PCOS [146].

**Figure 4:**
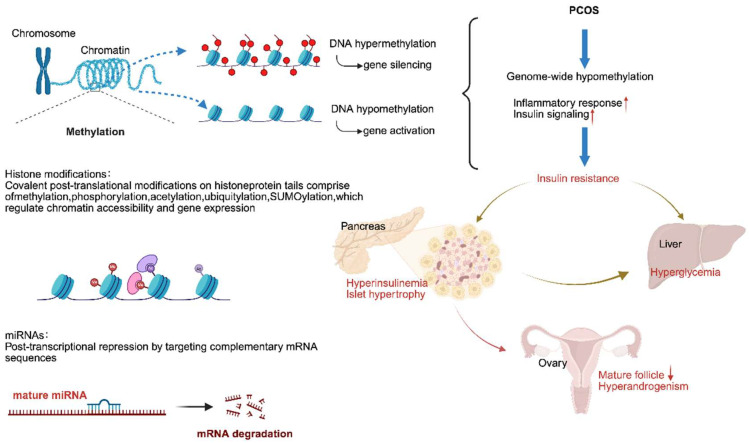
Interplay between epigenetic modifications in miRNA regulation in PCOS. A schematic diagram illustrating key mechanisms of gene expression regulation at the chromatin and post-transcriptional levels, and their association with metabolic dysregulation, as well as post-transcriptional control by microRNAs through RISC-guided translational repression or mRNA degradation. This diagram further links dysregulated epigenetic and miRNA networks to metabolic phenotypes such as pancreatic hyperinsulinemia with islet hypertrophy and hepatic hyperglycemia arising from impaired insulin signaling. Abbreviations: miRNA, microRNA; mRNA, messenger RNA. Blue dashed: Methylation → gene silencing/activation. Arrows: blue solid: PCOS → hypomethylation → insulin resistance; black solid: Histone/miRNA regulation; red: ↑ pathology, ↓ follicles. Gold solid: Cyclic metabolic links. ↑, Increase; ↓, Decrease. This figure was created in BioRender. Eva, L. (2026) https://BioRender.com/v09q89w (accessed on 23 February 2026).

Tissue-specific epigenetic alterations further underscore the complexity of PCOS pathogenesis. In ovarian granulosa cells, TET-mediated DNA demethylation dysregulates AMH in PCOS [147]. In ovarian theca cells, key steroidogenesis genes such as CYP11A1 and CYP17A1 are under epigenetic control in PCOS [23]. In adipose tissue, ADIPOQ hypermethylation reduces adiponectin expression and impairs insulin sensitivity [148]. Skeletal muscle also shows systemic metabolic dysfunction via DNA methylation remodeling of GLUT4 [149] (Figure 4).

Therapeutic approaches targeting epigenetic regulation, such as DNA methyltransferase inhibitors 5-azacytidine, restore aberrantly silenced gene expression [150]. Similarly, HDAC inhibitors like sodium butyrate (NaBu) have shown promising effects in PCOS animal models, including improvements in insulin sensitivity, hormonal balance, and estrus cycle restoration [151]. Natural products, including resveratrol, quercetin, curcumin, EGCG, and formononetin, exhibit anti-inflammatory and metabolic benefits through epigenetic and transcriptional modulation [152,153]. Furthermore, lifestyle factors like exercise and dietary modifications may restore metabolic and reproductive homeostasis via exercise-induced DNA methylation changes [154,155]. Epigenetic alterations, including DNA methylation, histone modifications, and ncRNAs, play a pivotal role in the pathogenesis of PCOS by driving ovarian dysfunction and metabolic abnormalities. These changes are dynamic, tissue-specific, and responsive to environmental factors, making them potential biomarkers for diagnosis and targets for precision therapy (Table 4).

### 2.5. Mechanisms of Natural Products Against Gut Microbiota Dysbiosis in PCOS

The gut microbiota shapes the intestinal environment and functions as an endocrine organ with systemic metabolic effects [159,160], and patients with PCOS exhibit distinct gut microbiota communities compared to healthy controls [161,162]. Gut microbiota dysbiosis contributes to IR, hyperandrogenism, chronic inflammation through multiple interconnected mechanisms including production of short chain fatty acids (SCFAs) and lipopolysaccharides (LPSs), sex hormone modulation, and brain–gut axis signaling [163,164]. Microbial imbalance disrupts metabolite production, leading to adipokine dysregulation and inflammation in adipose tissue, which impairs insulin signaling and pancreatic β-cell dysfunction [165,166]. Bile acid metabolism represents an additional pathway. Reduced IL-22 in PCOS promotes the increase in bacilli in intestines, altering bile acid synthesis and aggravating IR [167,168]. Dysbiosis-induced release of intestinal endopeptidase, cytokines, and inflammatory mediators triggers systemic low-grade inflammation, exacerbating hyperandrogenism, IR, and ovulatory dysfunction [169,170]. Furthermore, gut microbiota composition correlates positively with androgen levels and negatively with estradiol, suggesting bidirectional interactions between the microbiome and reproductive hormone homeostasis [171,172,173].

**Figure 5:**
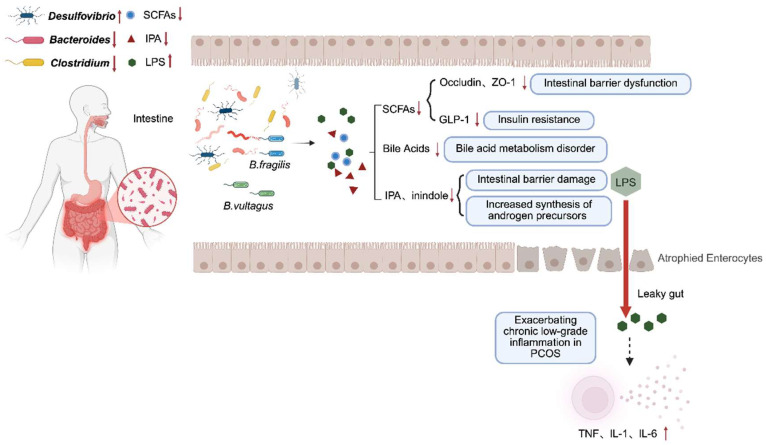
Proposed mechanisms linking gut microbiota dysbiosis to PCOS pathophysiology. Altered gut microbiota (dysbiosis) contributes to PCOS by reducing SCFA- and IPA-producing bacteria, increasing LPS-producing taxa, impairing intestinal barrier integrity (Occludin, ZO-1), and promoting systemic inflammation, IR, and androgen excess. Abbreviations: GLP-1, glucagon-like peptide-1; IPA, indole-3-propionic acid; LPS, lipopolysaccharide; PCOS, polycystic ovary syndrome; SCFAs, short-chain fatty acids; ZO-1, zonula occludens-1. Arrows: **↑**, Increase (*Desulfovibrio*, LPS). **↓**, Decrease (*Bacteroides*, *Clostridium*, SCFAs, IPA). Colors: Blue (*Desulfovibrio*, SCFAs); Red (*Bacteroides*, IPA); Yellow (*Clostridium*); Green (LPS). Shapes: Circle (SCFAs); Triangle (IPA); Hexagon (LPS). This figure was created in BioRender. Eva, L. (2026) https://BioRender.com/976c3qp (accessed on 23 February 2025).

Gut microbiome alterations affect follicle development, oocyte maturation, and embryo migration [174]. In PCOS models, fecal microbiota transplantation (FMT) reduces androgens, restores estrous cycles, and improves ovarian function [172], while antibiotics improve glucose tolerance and insulin sensitivity [175,176,177]. Mechanistically, the bacterial metabolite agmatine ameliorates ovarian dysfunction via FXR-GLP-1 signaling and intestinal hypoglycemic hormones including glucagon-like peptide-1 (GLP-1), gastric inhibitory peptide (GIP), and growth differentiation factor 15 (GDF15), linking the gut microenvironment to systemic metabolism and reproductive homeostasis [178,179,180]. PCOS pathophysiology extends beyond the HPO axis to involve gut–brain communication, mediated by vagal nerve activation and neurotransmitter/peptide signaling [181,182,183]. Thus, gut microbiota dysbiosis affects follicular development, reproductive hormone balance, and metabolism through interconnected pathways involving IR, hyperandrogenism, chronic inflammation, and the brain–gut axis, positioning it as a critical nexus in PCOS pathogenesis and a promising therapeutic target (Figure 5).

Accumulating evidence highlights that dietary bioactive compound, particularly polyphenols, flavonoids, and prebiotics derived from herbs and edible plants, exert therapeutic effects on PCOS primarily through modulation of the gut microbiota [184]. For instance, berberine enhances SCFA-producing bacteria such as *Blautia* and *Allobaculum*, activates AMPK signaling, and alleviates metabolic disturbances [185]. Quercetin increases microbial diversity and promotes beneficial taxa including *Lactobacillus* and *Bifidobacterium* [186]. Curcumin improves inflammatory and metabolic profiles in PCOS patients while beneficially altering gut microbiota and protecting ovarian function in experimental models [187,188]. Similarly, Astragalus polysaccharides (APS) mitigate IR and oxidative stress while increasing microbial diversity in PCOS mice [189]. Beyond individual compounds, probiotic and symbiotic bacteria, including *Lactobacillus* and *Bifidobacterium* strains, have been validated in meta-analyses and RCTs to restore hormonal balance, reduce inflammation, and improve IR in women with PCOS [190,191] (Table 5). Collectively, these findings underscore that natural products ameliorate PCOS progression via gut microbiota-dependent mechanisms, positioning them as promising candidates for integrative therapeutic strategies.

In summary, PCOS pathophysiology is multifactorial, involving endocrine disruption, IR, oxidative stress-induced cell death, epigenetic modifications, and gut microbiota dysbiosis. Hyperandrogenism, largely driven by aberrant HPO axis signaling, enhances LH responsiveness and dysregulates steroidogenesis, thereby impairing folliculogenesis and ovulation. IR synergistically reinforces androgen excess and metabolic disturbances, while oxidative and ER stress accelerates granulosa cell apoptosis and ferroptosis, exacerbating ovarian dysfunction. Epigenetic remodeling links genetic and environmental triggers, perpetuating hormonal and metabolic imbalance. The gut microbiota further modulates androgen levels, insulin sensitivity, and the brain–gut axis. These interconnected mechanisms establish a vicious cycle of reproductive and metabolic derangements, necessitating multi-targeted therapeutic strategies. Natural products, with pleiotropic actions on these pathways, offer promising integrative approaches to restore homeostasis in PCOS.

## 3. A Metabolic–Reproductive Axis Perspective on Natural Products for PCOS

Metabolic dysfunction represents a pivotal hub of PCOS, integrating dyslipidemia, obesity, and hormonal imbalances that perpetuate a vicious cycle between metabolic and reproductive disturbances. Dyslipidemia and obesity exacerbate IR and impair reproductive outcomes. Menstrual dysfunction serves as a cardiometabolic risk, linking ovarian dysregulation to systemic complications such as hypertension, dyslipidemia, and cardiovascular disease. Hormonal imbalances involving insulin, gonadotropins, adipokines, and androgens contribute synergistically to the process of PCOS, underscoring the metabolic–reproductive axis as a critical therapeutic target.

### 3.1. Targeting Hormonal Drivers of the Metabolic-Reproductive Axis

Hormonal dysregulation is a cornerstone of PCOS pathophysiology, involving disruptions in insulin, the anti-Müllerian hormone (AMH), and other endocrine regulators [195,196]. Hyperandrogenism, a hallmark of PCOS, arises from the dysregulation of the HPO and adrenal axis and is further exacerbated by hyperinsulinemia [197]. Beyond its origins, prenatal androgen overexposure has been implicated in endocrine and metabolic dysfunctions in the fetus stage, increasing the risk of associated metabolic diseases [198,199]. Conversely, excess insulin can elevate androgen levels. Theca cells in women with PCOS are more susceptible to insulin-stimulated androgen synthesis compared to those from healthy women [200]. This bidirectional insulin–androgen interaction establishes a self-perpetuating cycle in the metabolic–reproductive axis. Furthermore, elevated androgens induce inflammation and pyroptosis of granulosa cells, thereby disrupting normal follicular development in PCOS patients [201].

The anti-Müllerian hormone (AMH), secreted by granulosa cells, serves as a reliable indicator of ovarian reserve and gonadotropin responsiveness [202]. It inhibits aromatase activity, reducing the conversion of androgens to estrogens and perpetuating hyperandrogenism. Elevated AMH levels in PCOS, resulting from increased antral follicle counts, correlate strongly with the polycystic ovarian morphology (PCOM) phenotype and reduced ovulatory rates [203,204]. Collectively, these interconnected hormonal drivers, including insulin, androgens, and AMH, represent promising therapeutic targets for restoring metabolic–reproductive axis homeostasis in PCOS [205,206].

### 3.2. Restoring Menstrual Function via Axis Modulation

Menstrual dysfunction in PCOS is both a core clinical feature and a biomarker of metabolic disturbance. The severity of menstrual irregularity from oligomenorrhea to amenorrhea correlates positively with the degree of IR, central adiposity, and dyslipidemia [207,208]. Women with amenorrhea exhibit ~3.1-fold higher IR prevalence and worse cardiometabolic profiles [209,210]. Thus, menstrual severity may help identify PCOS subgroups at elevated metabolic risk.

Mechanistically, hyperandrogenism disrupts folliculogenesis and HPO axis signaling. Obesity-related inflammation and adipose IR further promote ovarian androgen excess and anovulation [211,212,213]. Hyperinsulinemia stimulates theca cell steroidogenesis and reduces SHBG, increasing bioavailable androgens. This reciprocal interaction defines a metabolic–reproductive axis in PCOS, wherein menstrual recovery reflects systemic metabolic improvement.

Beyond reproduction, the cumulative metabolic burden of IR, dyslipidemia, oxidative stress, endothelial dysfunction, and chronic inflammation elevates long-term CVD risk in women with PCOS, with anovulatory phenotypes exhibiting more pronounced atherogenic lipid perturbations and heightened cardiometabolic vulnerability [214,215]. PCOS women show increased cardiovascular events, and venous thromboembolism [216,217], early-life hyperandrogenism, and metabolic dysfunction may accelerate risk accumulation in PCOS, yet postmenopausal data remain scarce.

Therefore, therapies targeting integrated metabolic–reproductive modulation to improve insulin sensitivity, reduce androgens, and alleviate inflammation may restore ovulation and mitigate cardiometabolic risk. Long-term cohort studies are needed to assess whether early intervention alters lifetime cardiovascular outcomes in PCOS.

### 3.3. Ameliorating Dyslipidemia and Obesity Through Axis-Targeted Intervention

Given the intricate correlation between metabolic dysfunction and reproductive disorders in PCOS, interventions targeting the metabolic–reproductive axis have emerged as a rational therapeutic strategy. Dyslipidemia, the most prevalent metabolic abnormality in PCOS, affects 41.3% of Chinese patients and is characterized by low HDL with elevated LDL and TG, which not only increases cardiovascular risk but also directly impairs reproductive outcomes [218,219,220]. Similarly, obesity affects 35–60% of women with PCOS and exacerbates key phenotypes such as menstrual irregularities and hyperandrogenism [221]. Notably, nearly 42% of patients exhibit abdominal obesity with visceral fat accumulation, linked to low-grade inflammation, reduced adiponectin, and IR [222,223]. Collectively, metabolic dysfunction including dyslipidemia and obesity drives PCOS pathogenesis and progression.

The metabolic–reproductive axis is characterized by directional crosstalk. Circulating adiponectin sourced from subcutaneous adipose tissue in PCOS may be dysfunctional and is correlated with IR [224]. These metabolic disturbances operate bidirectionally within the axis. Adipose tissue-specific IR impairs free fatty acid (FFA) suppression, and elevated FFAs directly stimulate ovarian androgen production, while hyperandrogenemia perpetuates IR and visceral adiposity, forming a self-reinforcing cycle that may begin prepubertally and progress toward the adult PCOS phenotype [198,225].

Consequently, early and effective weight management forms the foundation for metabolic–reproductive axis modulation. Bariatric surgery reduces adiposity and mitigates both infertility and cardiovascular risk [226,227]. Conventional pharmacologic approaches, including insulin sensitizers, lipid-lowering agents, and anti-androgens, target discrete components of the axis. Notably, natural products exert multi-target effects by simultaneously ameliorating IR, attenuating androgen excess, and improving lipid profiles with favorable safety profiles. By addressing multiple nodes of the axis, natural products represent promising adjuncts or core components of future integrated therapeutic strategies for PCOS.

## 4. Innovative and Emerging Therapeutic Strategies of Natural Products in PCOS

PCOS is a multifaceted disorder with reproductive, metabolic, and psychological consequences that extend far beyond its traditional clinical features. Increasing recognition of its heterogeneity has driven a shift from conventional symptom-oriented treatments toward more integrative and mechanism-based approaches. Current evidence highlights that no single therapy adequately addresses the complex interplay of IR, hyperandrogenism, and mood disturbances in PCOS. Consequently, innovative therapeutic strategies, particularly phytochemical-based therapies, alongside bariatric surgery, lifestyle interventions, pharmacological regimens, nutritional supplementation, phytochemicals, and psychological care, are emerging as critical components of a comprehensive management framework. Natural products, owing to their multi-targeting properties and favorable safety profiles, have garnered particular interest in addressing the metabolic and endocrine disturbances central to PCOS. Moreover, novel biomarkers such as circulating hedgehog-interacting protein (HHIP) are opening avenues for precision medicine. Collectively, these strategies represent a paradigm shift toward holistic, patient-centered, and multidisciplinary care. With natural products assuming a central role, this integrative approach marks a significant advance in PCOS treatment.

### 4.1. The Metabolic–Endocrine Axis in PCOS: A Rationale for Natural Product Intervention

PCOS is fundamentally characterized by bidirectional dysregulation of the metabolic–endocrine axis. IR drives compensatory hyperinsulinemia, which stimulates ovarian androgen production and suppresses hepatic SHBG synthesis, elevating free androgens. Hyperandrogenemia, in turn, exacerbates visceral adiposity and impairs insulin sensitivity, creating a self-perpetuating cycle central to PCOS heterogeneity.

Current interventions target this axis incompletely. Bariatric surgery offers notable metabolic and reproductive benefits including reducing body mass index (BMI), restoring menstrual irregularities and hirsutism, normalizing androgens, and improving fertility [228,229,230]. However, it is invasive and has potential risks including nutrient deficiencies and unfavorable pregnancy outcomes; thus, it is reserved for refractory cases [231]. COCs effectively manage hyperandrogenism but may exacerbate IR and hepatic fat accumulation [232,233,234]. Even within multidisciplinary, preventive care frameworks—incorporating regular metabolic surveillance and coordinated specialist input—a critical gap persists: no single agent safely addresses both metabolic and endocrine disturbances simultaneously.

Natural products fill this gap. Their inherent multi-targeting properties enable concurrent modulation of insulin sensitivity, androgen excess, inflammation, and oxidative stress [18,32]. By acting on insulin signaling, steroidogenesis, and inflammatory cascades, they offer potential to restore metabolic–endocrine homeostasis. This rationale underpins the following sections examining natural product strategies in PCOS management.

### 4.2. Lifestyle and Behavioral Modification: Foundations of Metabolic–Endocrine Modulation in PCOS

Weight loss of just 5% of initial body weight significantly improves insulin sensitivity, reproductive function, and hormonal balance in PCOS [235]. Structured exercise combined with dietary modification, particularly involving low-glycemic-index carbohydrates, high fiber, healthy fats, and lean proteins, enhances insulin action, reduces androgen excess, and restores ovulatory cycles [236]. Regular physical activity, especially aerobic and resistance training, improves metabolic markers. A 20-week home-based aerobic exercise program reduced HOMA-IR and inflammation in women with PCOS [237]. Combined diet and exercise result in better reproductive and metabolic outcomes compared to either intervention alone [238,239].

Crucially, these lifestyle modifications create a physiological foundation that optimizes subsequent natural product interventions. By improving insulin sensitivity, reducing inflammation, and restoring hormonal balance, lifestyle changes establish a permissive metabolic–endocrine environment for natural products to exert their multi-targeted effects more effectively. Enhanced insulin sensitivity amplifies the roles of insulin-sensitizing agents like berberine or inositol, while reduced oxidative stress primes cellular pathways for antioxidants such as resveratrol and curcumin. Thus, lifestyle modification is not an alternative to natural product strategies but an indispensable foundation maximizing therapeutic efficacy in PCOS management.

### 4.3. IR as the Metabolic–Endocrine Nexus in PCOS

IR represents a critical interface between metabolic and endocrine disturbances in PCOS, driving a self-perpetuating cycle wherein hyperinsulinemia stimulates ovarian androgen production, suppresses hepatic SHBG synthesis, and potentiates LH-driven theca cell steroidogenesis, while hyperandrogenism in turn promotes visceral adiposity and further metabolic dysfunction. Lifestyle modification remains foundational, but pharmacological options like metformin are often limited by gastrointestinal side effects and variable efficacy, driving interest in natural products as safer, multi-targeted alternatives [240]. Beyond their direct insulin-sensitizing effects detailed in Section 2.2, natural products modulate this axis through integrative mechanisms, including epigenetic regulation, gut microbiome modulation, and SIRT1-mediated oxidative stress reduction, that address both metabolic and endocrine components [241,242,243,244]. Thus, natural products target IR not merely as a metabolic disturbance but as the central node connecting metabolic dysfunction to endocrine dysregulation, offering a conceptual framework for multi-targeted interventions in PCOS.

### 4.4. Regulating Androgen Excess: Natural Products as Endocrine Modulators

Hyperandrogenism is a central endocrine disturbance in PCOS, arising from ovarian theca cell hyper-responsiveness to LH, amplified by hyperinsulinemia, which stimulates androgen synthesis and suppresses hepatic SHBG, increasing bioavailable testosterone. Conventional pharmacotherapies such as oral contraceptives, anti-androgens, and insulin sensitizers are constrained by teratogenicity, hepatotoxicity, adverse metabolic effects, or contraceptive requirements, prompting interest in safer natural alternatives [245,246].

Natural products regulate androgen excess via three mechanisms. First, they inhibit steroidogenic enzymes including ovarian CYP17A1 and 5α-reductase, reducing androgen synthesis and peripheral conversion. Second, they upregulate hepatic SHBG, reducing free androgens. Third, they enhance insulin sensitivity, attenuating insulin-driven theca cell androgen production and disrupting metabolic–endocrine crosstalk [126,247,248,249]. Specific natural products exhibit androgenic regulation. Fenugreek improves hormonal profiles and reduces androgenic symptoms in women with dyslipidemia or alopecia [250,251]. Tinospora cordifolia and Garcinia cambogia restore ovarian function and hormonal balance [52,83]. Resveratrol and curcumin suppress ovarian androgen production via anti-inflammatory and antioxidant pathways that mitigate theca cell hyperstimulation [32,218].

### 4.5. Synergistic Strategies: Integrating Nutritional Supplements with Natural Products

Nutritional supplements have emerged as valuable adjuncts targeting IR, hyperandrogenism, and reproductive dysfunction that persist despite lifestyle and pharmacotherapy [252,253]. Evidence-based micronutrients and bioactive compounds, such as vitamin D, inositol isomers (myo-inositol and D chiro inositol), omega-3 fatty acids, NAC, and antioxidants, improve metabolic parameters, hormonal balance, and oxidative stress [254]. These supplements exhibit mechanistic complementarity with natural products. Myo-inositol enhances insulin signal transduction, potentially synergizing with berberine-induced AMPK activation or resveratrol-mediated SIRT1 upregulation to amplify insulin sensitization. Myo-inositol alone improves fasting insulin, HOMA-IR, and SHBG, mitigating hyperandrogenism [252,255]. Vitamin D enhances glycemic control, chromium, and inositol. Omega-3 fatty acids favorably modulate lipid profiles, hormonal regulation, and inflammation [249,252]. This combination of nutritional supplements and natural products targets IR, androgen excess, oxidative stress, and inflammation through complementary pathways, offering broader therapeutic coverage aligned with PCOS heterogeneity [256].

### 4.6. A Holistic Perspective: Psychological Well-Being in PCOS Care

Psychological well-being is a vital, and often underacknowledged, consideration in the holistic treatment of women with PCOS. Mood disorders, especially anxiety and depression, are highly prevalent in PCOS, with psychiatric morbidity affecting approximately 50% of women with PCOS, including anxiety disorders at 38.6% and depressive disorders at 25.7% [257,258,259]. Depressive symptoms affect around 31–37% of women with PCOS, and anxiety symptoms are common as well [260,261]. These mood disturbances stem from hormonal imbalances and IR, as well as the visible manifestations of PCOS, such as hirsutism, acne, obesity, and infertility, which undermine body image and self-esteem [262,263,264]. These associations necessitate integrated therapeutic approaches.

Natural products offer unique advantages in PCOS care. For example, resveratrol, curcumin, and quercetin alleviate metabolic-driven mood disturbances by improving IR and inflammation. Select botanicals possess direct neuroactive properties: saffron exerts antidepressant effects, and ashwagandha modulates cortisol and anxiety. Integrating psychological support with natural product-based strategies thus enables truly holistic care, improving emotional well-being while enhancing adherence to therapeutic interventions and optimizing long-term outcomes.

### 4.7. A New Paradigm: Continuous Monitoring and Multidisciplinary Care

In order to optimize therapeutic outcomes of natural products in PCOS, it will be necessary to continuously monitor and collaborate in a multidisciplinary manner. Given the heterogeneous nature of PCOS, treatment must be carefully tailored to each patient’s specific clinical presentation, symptom severity, and reproductive or metabolic goals. No single therapeutic approach is universally effective. Personalized care plans should consider factors such as age, body mass index, fertility intentions, psychological status, and comorbidities. A multidisciplinary, collaborative approach is strongly recommended. Coordination among endocrinologists, gynecologists, nutritionists, mental health professionals, and, when necessary, bariatric specialists ensure a more comprehensive and holistic model of care. This team-based strategy facilitates the integration of medical, nutritional, lifestyle, and psychosocial interventions, ultimately improving the long-term health outcomes and quality of life for women living with PCOS. The evolving concept of PCOS management emphasizes not only symptomatic treatment but also continuous, dynamic monitoring as a novel therapeutic strategy. Rather than relying on static evaluations, regular surveillance of metabolic markers (e.g., fasting glucose, insulin, lipids, body composition) and reproductive hormones enables early detection of complications such as type 2 diabetes, cardiovascular risk, and progressive hyperandrogenism. This forward-looking approach supports proactive interventions, allowing timely adjustment of treatment regimens before irreversible damage occurs. Given the heterogeneity of PCOS, individualized care plans that integrate reproductive goals, age, BMI, psychological health, and comorbidities are increasingly recognized as essential. A multidisciplinary and collaborative model linking endocrinology, gynecology, nutrition, mental health, and bariatric expertise represents a new paradigm of care. By integrating medical, nutritional, lifestyle, and psychosocial support, this innovative approach shifts PCOS management from reactive to preventive, ultimately improving long-term health and quality of life for affected women.

### 4.8. Novel Biomarkers: Toward Precision Diagnosis and Therapy

In recent years, advances in omics technologies and molecular biology have identified several novel biomarkers that may provide deeper insights into the metabolic, reproductive, and endocrine disturbances of PCOS. These emerging biomarkers not only improve diagnostic precision but also offer potential targets for personalized treatment strategies.

Serum HHIP levels are elevated in PCOS with IR, correlating with obesity, metabolic dysfunction, and reproductive hormone changes [265]. Glycolysis-associated metabolites are also altered, directly linking abnormal glucose metabolism to pathogenesis [266]. These metabolic biomarkers are suitable for insulin-sensitizing natural products. Additionally, HDDC3 and SDC2 in granulosa cells were identified as potential diagnostic biomarkers for PCOS (AUC > 0.8) correlating with immune cell infiltration, suggesting roles in PCOS immune regulation [267]. Serum markers including Visfatin, IGF1, IGFBP1, SREBP1, and Salusin-β are altered in PCOS and may be linked to increased risk of endometrial or ovarian malignancy. These proteins represent emerging biomarkers for cancer risk stratification in PCOS [268]. Moreover, proteomic studies revealed changes in ovarian proteins, including upregulated PGRMC1, RBP1, HSP90B1, and CALM1 and downregulated ANXA6 and TPM2 in PCOS. These proteins are involved in proliferation, endocrine regulation, inflammation, and thrombosis, highlighting their potential as mechanistic biomarkers [269,270]. Computational analysis identified upregulated HSPA5, PLK1, RIN3, DBN1, and CCDC85 and downregulated DISC1, AR, MTUS2, LYN, and TCF4 hub genes in PCOS. These genes are enriched in pathways related to development, interferon response, and endocrine regulation, providing new molecular biomarkers [271] that are suggested to guide rational selection of natural products targeting specific dysregulated pathways.

Together, these metabolic, immune, endocrine, and oncogenic biomarkers underscore the complexity of PCOS. Future large-scale, longitudinal, and multi-ethnic studies are essential to validate these candidates and to translate them into clinical practice. Ultimately, novel biomarkers may enable more precise phenotyping of PCOS and foster personalized treatment approaches tailored to individual metabolic and reproductive profiles.

Natural products are emerging as core components of PCOS therapy through their ability to modulate the metabolic–endocrine axis by targeting IR, androgen excess, inflammation, and oxidative stress via complementary pathways including AMPK activation, SIRT1 modulation, and gut microbiota regulation. Natural products and nutritional supplements offer multi-targeted benefits with favorable safety profiles. These effects are potentiated by foundational lifestyle interventions and optimized within continuous monitoring and multidisciplinary care frameworks. Emerging biomarkers further enable precise phenotyping, guiding selection of natural products matched to individual metabolic–endocrine profiles. Together, these advances establish a new paradigm for integrative, mechanism-driven PCOS management centered on natural product-mediated restoration of metabolic–endocrine homeostasis.

## 5. Conclusions

Current research on natural products for PCOS focuses on improving IR, hormonal imbalance, inflammation, and oxidative stress. Among these, inositols (myo-inositol and D-chiro-inositol) show the strongest clinical evidence for improving insulin sensitivity, restoring ovulation, and regulating cycles. Berberine exhibits metabolic effects comparable to metformin, and N-acetylcysteine (NAC) enhances antioxidant capacity and insulin signaling, supporting ovulatory function. Other compounds, including quercetin, resveratrol, and formononetin, show potential in modulating inflammation and ovarian signaling, though clinical evidence remains preliminary. Cinnamon, spearmint, *Vitex agnus-castus*, and licorice are primarily used for hyperandrogenism or metabolic abnormalities. Overall, these are adjunctive options with varying evidence.

Future research will increasingly target ovarian fibrosis, chronic low-grade inflammation, mitochondrial dysfunction, and the gut–ovarian axis, which are central pathological features of PCOS. Isoflavones such as formononetin and genistein may modulate estrogen receptors. Triterpenoids/saponins derived from *Astragalus*, *Dioscorea*, and *Gynostemma* exhibit anti-fibrotic and metabolic effects; polyphenols, including curcumin derivatives, EGCG, and flavonoids, act through modulation of key signaling pathways such as TGF-β/Smads, AMPK, and NF-κB. Polyphenols also enhance SCFA production via gut microbiota, strengthening the intestinal barrier and reducing inflammation. Prebiotics, such as fructooligosaccharides (FOSs), inulin, and galactooligosaccharides (GOSs), promote the proliferation of *Bifidobacterium,* maintaining gut microbial homeostasis and reinforcing the intestinal barrier.

Natural products offer multi-target effects, favorable safety, and suitability for long-term use, modulating IR, chronic inflammation, oxidative stress, and hormonal imbalance. They are beneficial as adjunctive interventions, particularly for moderate metabolic-dominant PCOS phenotypes. However, critical limitations remain, as current evidence is largely based on small-scale clinical studies or preclinical data, with challenges including insufficient standardization, variable bioavailability, delayed onset of action, and limited therapeutic potency. Addressing these issues requires high-quality clinical trials and standardized quality control clinical trials, as well as advanced delivery technologies and precision strategies based on PCOS phenotypes and biomarkers to enhance bioavailability and therapeutic efficacy. Integration with lifestyle interventions and conventional treatments, aligned with international standards, will support safe and evidence-based clinical application.

Bridging preclinical promise and clinical application is the central challenge. Future research should prioritize these aspects such as through dose–response studies, combination therapies, long-term safety evaluations, advanced delivery systems, and precision strategies based on PCOS phenotypes. Integration with lifestyle and conventional treatments, aligned with international standards, is essential.

In summary, PCOS is a systemic and heterogeneous disorder arising from genetic susceptibility, endocrine dysregulation, IR, low-grade inflammation, and environmental factors. These processes disrupt ovarian steroidogenesis, folliculogenesis, and ovulation while increasing cardiometabolic and psychological risk. Therapies targeting single pathways rarely achieve durable control, whereas natural products show translational potential by modulating core pathogenic mechanisms such as IR, chronic inflammation, oxidative stress, fibrosis, and dysregulated signaling, including AMPK, PI3K-Akt, TGF-β/Smads, and NF-κB with low toxicity. Their multi-target and systemic regulatory properties modulate the metabolism–inflammation–endocrine network, targeting key mechanisms like the gut–ovarian axis and ovarian fibrosis, and supporting long-term, safe metabolic and reproductive health. By reshaping physiological homeostasis through precise, multi-pathway interventions, natural products provide a foundation for individualized, full-cycle prevention and improvement in the long-term outcomes of PCOS. Realizing this potential, however, will require a concerted effort to translate mechanistic insights into clinically validated therapies through rigorous, well-designed human studies.

## Figures and Tables

**Table 1:** Natural products targeting hormonal dysregulation in PCOS (↑ upregulation; ↓ downregulation).

Natural Product	Source	Effects and Mechanisms	References
Coenzyme Q10	*Oryza sativa*, *Triticum aestivum*	↑ SHBG and ↓ testosterone in RCT	[34]
Epigallocatechin-3 gallate (EGCG)	*Camellia sinensis*	↓ free testosterone (FT) and FSH levels in PCOS patients	[35]
Flaxseed	*Linum usitatissimum*	↑ FSH, ↓ LH/FSH ratio, ↓ Total testosterone, and ↑ SHBG levels in RCT	[36,37]
Inositols	*Fabaceae*	Increasing SHBG levels in RCT	[38]
Isoflavone	*Glycine max*	↓ testosterone in RCT	[39,40]
Mentha arvensis extract	*Mentha arvensis*	↓ Cyp17 and Ptgs2 expression, ↑ antioxidant capacity, and ↓ numbers of cysts in PCOS rats	[41]
Oregano Essence	*Origanum vulgare*	Balancing GnRH, FSH, and LH levels in PCOS rat	[42]
Resveratrol	*Polygonum cuspidatum*	↓ serum testosterone and FSH levels, ↓ glycolysis and normalizing HPO axis dynamics in PCOS rats	[43,44,45]
Shatavari	*Asparagus racemosus*	↓ oxidative stress, ↑ menstrual regularity and support HPO axis in PCOS women	[46,47]
Stinging Nettle	*Urtica dioica*	↓ total and free testosterone in PCOS mice	[48]
Tinospora cordifolia	*Tinospora cordifolia*	Balancing LH and FSH levels in PCOS mice	[49]
Vitamin D	Mushroom	↑ LH/FSH ratio, ↓ total testosterone (TT) and ↑ SHBG levels in RCT	[50,51]
Vitex agnus-castus extract	*Vitex agnus-castus*	↓ testosterone and LH, ↑ FSH and progesterone and adjusting the HPG axis through KISS-1 gene in PCOS rats	[52,53]

**Table 2:** Natural products for targeted regulation of IR in PCOS (↑ upregulation; ↓ downregulation).

Natural Product	Source	Effects and Mechanisms	References
Alpha-Lipoic Acid	*Spinacia oleracea, Brassica oleracea, Solanum lycopersicum*	↑ the uptake of glucose in liver, adipose tissue, skeletal muscle, and ovaries in PCOS patients	[68]
Berberine	*Hydrastis canadensis*	↑ HOMA, visceral adipose tissue, and fat mass in PCOS patients	[69,70]
Coenzyme Q10	*Spinacia oleracea*	↓ HOMA-IR, ↓ fasting insulin, ↓ fasting glucose in RCT	[34,71]
Cinnamon	*Cinnamomum verum*	↑ insulin sensitivity in RCT	[72,73,74,75]
Curcumin	*Curcuma longa*	↓ blood sugar levels and IR in RCT	[76,77]
Gymnemic Acid	*Gymnema sylvestre*	Delaying glucose absorption in PCOS patients	[78]
Inulin-type fructans	*Cichorium intybus, Helianthus tuberosus, Allium sativum*	Improving IR and hyperandrogenemia in RCT	[79]
*N*-acetylcysteine (NAC)	*Allium sativum*, *Allium cepa*	Improving endocrine–metabolism profiles in PCOS mice	[80,81]
Omega-3	*Salvia hispanica*	↓ FPG and inflammatory factors, ↑ blood lipid metabolism, and ↓ IR in a meta-analysis of RCT	[82,83]
Quercitrin	*Albizia julibrissin*	↑ IR, ↓ lipogenesis, and ↑ PM20D1 and PI3K/Akt pathway in PCOS rats	[84]
Resveratrol	*Polygonum cuspidatum*	Activating SIRT2 and improving glycolytic pathway in PCOS rats	[85]
Stinging Nettle	*Urtica dioica*	↑ insulin sensitivity in PCOS mice	[48,86]
Tinospora cordifolia	*Tinospora cordifolia*	↑ insulin sensitivity in PCOS mice	[87]

**Table 3:** Natural products for targeted regulation of oxidative stress and apoptosis in PCOS (↑ upregulation; ↓ downregulation).

Natural Product	Source	Effects and Mechanisms	References
Alpha-Lipoic Acid	*Spinacia oleracea*, *Brassica oleracea*, *Solanum lycopersicum*	Modulating apoptosis nodes (BAX/Bcl-2/Caspase-3) and NF-κB-linked inflammatory–apoptotic coupling Activating Nrf2 and ↓ apoptosis in meta-analysis of RCT	[110,111]
Astaxanthin	*Haematococcus pluvialis*	↑ antioxidant capacity (TAC, CAT), ↓ ovarian cysts, apoptosis, and necrosis, ↓ serum OS markers (MDA, SOD and TAC) and ER stress (GRP78, CHOP, XBP1), ↓ inflammatory cytokines (IL-6, IL-18, TNF-α) in RCT	[112]
Baicalein	*Scutellaria baicalensis* Georgi	↓glutathione peroxidase and ferritin heavy chain 1 and ferroptosis in PCOS rat	[113]
Bitter Melon extract	*Momordica charantia.*	↓ antioxidant markers SOD and CAT in PCOS rats	[114]
Cinnamon	*Cinnamomum verum*	↓ oxidative stress in PCOS patients	[115]
Curcumin	*Curcuma longa*	↑ PPAR-γ expression and ↓ oxidative stress in PCOS rat; ↓ IRE1α-XBP1 over-activation, ↓ GC apoptosis, and ↑ ovarian function in PCOS rats	[116,117]
Epigallocatechin-3-gallate	*Camellia sinensis, Hamamelis virginiana*	↑ antioxidant enzyme activity including SOD, catalase, glutathione reductase, PON-1 arylesterase, PON-1 CMPAase, etc.	[35]
Hydroxycitric acid	*Garcinia atroviridis, Garcinia cowa, Garcinia oblongifolia*	↓ oxidative stress (↓ MDA, ↑ SOD/CAT/GPx) in PCOS rat	[118]
Inulin-type fructans	*Cichorium intybus, Helianthus tuberosus, Allium sativum*	↑ serum levels of nitric oxide (NO), and ↓ endothelin-1 and total oxidant status in PCOS patients	[119]
Omega-3	*Salvia hispanica*	↓ serum GSH, MDA or TAC in PCOS patients	[120]
Palmitic acid	*Elaeis guineensis*	↑ the sensitivity of ferroptosis via endoplasmic reticulum stress mediated the ATF4/TXNIP axis in PCOS patients	[121]
Platycodin D	*Platycodon grandiflorum*	↑ CD44, ↓ ferroptosis in PCOS patients and rats	[122,123]
Quercetin	*Allium cepa*	↑ antioxidant enzyme activities, GSH levels, and ↓MDA levels and DNMT3a expression in PCOS rats; ↓ apoptosis in PCOS rats; ↓ TLR/NF-κB inflammatory–apoptotic coupling, and rebalancing BAX/Bcl-2/Caspase-3 in granulosa cells	[15,124,125,126,127,128,129]
Resveratrol	*Polygonum cuspidatum*, *Vitis vinifera*, *Gnetum parvifolium*, *Myristica fragrans*	↓ total oxidant status (TOS) and oxidative stress index (OSI), and ↑ expression of CAT and UCP2 in RCT	[130]
Saw Palmetto	*Serenoa repens*	↑ antioxidant defense SOD, catalase, and GSH in PCOS rat	[131]

**Table 4:** Natural products targeting epigenetic mechanisms in PCOS (↑ upregulation; ↓ downregulation).

Natural Product	Source	Effects and Mechanisms	References
EGCG	*Camellia sinensis*	↓ DNMT/HDAC in PCOS patients	[156,157]
Quercetin	*Allium cepa*	↓ DNMT, HDAC, and HMT, then activating and altering promoter CpG methylation and silencing genes in PCOS rats	[128,158]

**Table 5:** Natural products regulating gut microbiota dysbiosis in PCOS (↑ upregulation; ↓ downregulation).

Natural Product	Source	Effects and Mechanisms	References
Curcumin	*Curcuma longa*	Promoting the generation of SCFAs and regulating the intestinal flora (such as increasing Faecalibacterium prausnitzii)	[192]
Inulin-type fructans	*Cichorium intybus*, *Allium cepa*, *Allium sativum*	↑ the abundance of Actinobacteria, Fusobacteria, Lachnospira, and Bifidobacterium; ↓ the ratio of F/B and the abundance of proteobacteria, Sutterella, and Enterobacter; Improving obese PCOS women’s disease through the gut flora–inflammation–steroid hormone pathway.	[193]
Omega-3 supplements	*Linum usitatissimum*, *Salvia hispanica*	Improving gut microbiota dysbiosis, ↓ endotoxemia/inflammation	[194]

## Data Availability

No new data were created or analyzed in this study.

## Abbreviations

AMPK	AMP-activated protein kinase
PPARγ	Peroxisome proliferator-activated receptor gamma
AR	Androgen receptor
ACC	Acetyl-CoA carboxylase
AMH	Anti-Müllerian hormone
AS160	AKT substrate of 160 kDa
BMI	Body mass index
CBVs	Cotrending basis vectors
COCs	Combined oral contraceptives
CPT-1	Carnitine palmitoyltransferase I
DNMTs	DNA methyltransferases
DHEAS	Dehydroepiandrosterone sulfate
ECM	Extracellular matrix
ER	Endoplasmic reticulum
FSH	Follicle-stimulating hormone
FFAs	Free fatty acids
GLP-1	Glucagon-like peptide-1
GLUT4	Glucose transporter type 4
GS	Glycogen synthase
GSK3	Glycogen synthase kinase 3
GSV	GLUT4 storage vesicle
GnRH	Gonadotropin-releasing hormone
HCG	Human chorionic gonadotropin
HPO	Hypothalamic–pituitary–ovarian
HHIP	Hedgehog interacting protein
HOMA-IR	Homeostatic model assessment for insulin resistance
IL	Interleukin
IR	Insulin resistance
IPA	Indole-3-propionic acid
IRS-1	Insulin receptor substrate 1
LC3	Microtubule-associated proteins 1A/1B light chain 3B
LPS	Lipopolysaccharide
LH	Luteinizing hormone
MAPK	Mitogen-activated protein kinase
miR	microRNA
miRNA	microRNA
mRNA	messenger RNA
mTORC1	Mechanistic target of rapamycin complex 1
NF-κB	Nuclear factor kappa B
PCOS	Polycystic ovary syndrome
PI3K	Phosphoinositide 3-kinase
ROS	Reactive oxygen species
RISC	RNA-induced silencing complex
SCFAs	Short-chain fatty acids
SHBG	Sex hormone-binding globulin
SIRT1	Sirtuin 1
SOD	Superoxide dismutase
T2DM	Type 2 diabetes mellitus
TGF-β	Transforming growth factor-β
TNF-α	Tumor necrosis factor-α
TCA	Tricarboxylic acid
TSC1/2	Tuberous sclerosis complex 1/2
WAT	White adipose tissue
ZO-1	Zonula occludens-1
